# A Spatially Explicit Model of Synchronization in Fiddler Crab Waving Displays

**DOI:** 10.1371/journal.pone.0057362

**Published:** 2013-03-06

**Authors:** Sabrina Borges Lino Araujo, Ana C. Rorato, Daniela M. Perez, Marcio R. Pie

**Affiliations:** 1 Programa de Pós-Graduação em Ecologia e Conservação, Universidade Federal do Paraná, Curitiba, Paraná, Brazil; 2 Laboratório de Dinâmica Evolutiva e Sistemas Complexos, Departamento de Zoologia, Universidade Federal do Paraná, Curitiba, Paraná, Brazil; 3 Programa de Pós-Graduação em Zoologia, Universidade Federal do Paraná, Curitiba, Paraná, Brazil; The Australian National University, Australia

## Abstract

Fiddler crabs (*Uca* spp., Decapoda: Ocypodidae) are commonly found forming large aggregations in intertidal zones, where they perform rhythmic waving displays with their greatly enlarged claws. While performing these displays, fiddler crabs often synchronize their behavior with neighboring males, forming the only known synchronized visual courtship displays involving reflected light and moving body parts. Despite being one of the most conspicuous aspects of fiddler crab behavior, little is known about the mechanisms underlying synchronization of male displays. In this study we develop a spatially explicit model of fiddler crab waving displays using coupled logistic map equations. We explored two alternative models in which males either direct their attention at random angles or preferentially toward neighbors. Our results indicate that synchronization is possible over a fairly large region of parameter space. Moreover, our model was capable of generating local synchronization neighborhoods, as commonly observed in fiddler crabs under natural conditions.

## Introduction

The emergence of large-scale synchronization patterns from the collective behavior of interacting agents has become a major area of research in recent years. Biological phenomena as disparate as neuronal activation [1], [2], unison clapping in large audiences [3], [4], and the spatial synchronization of population cycles leading to outbreaks [5] have been shown to result from the individual responses of constituent parts without any centralized control [6], [7]. Understanding the mechanisms underlying such emergence, as well as the commonalities and differences between physical and biological self-organizing systems, can provide a valuable tool to assist in overcoming the limitations of traditional reductionistic approaches, and thus to contribute to a more comprehensive understanding of the origin and maintenance of biological diversity [8], [9].

**Figure 1:**
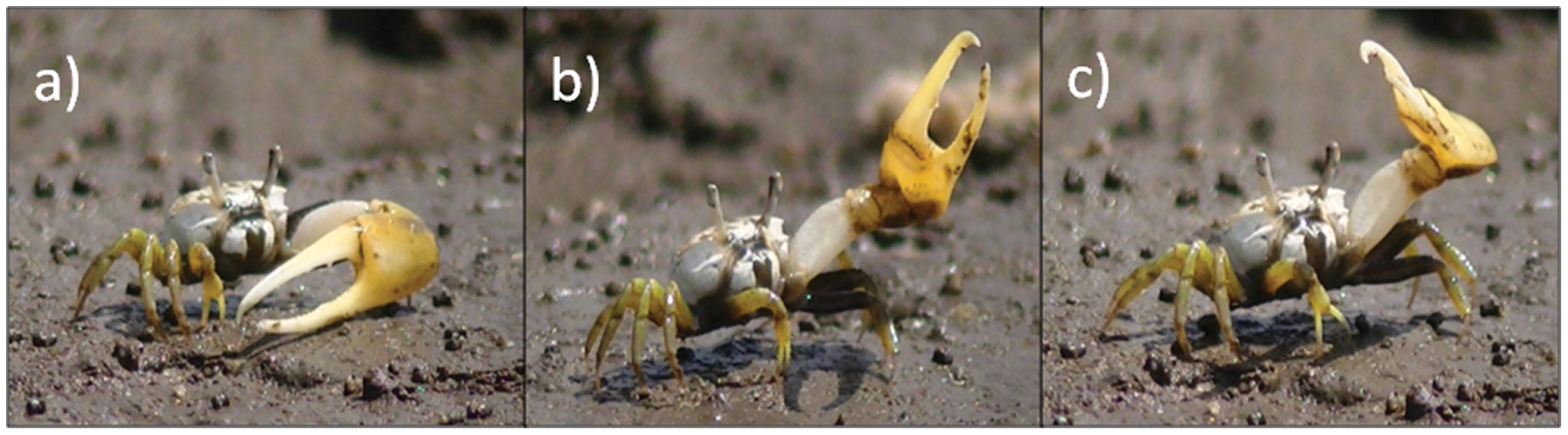
Waving-display in male of *Uca leptodactylus* illustrating fiddler crab waving behavior. (a) Initial upward movement; (b) waving apex; (c) final downward movement. Photo: Ana C. Rorato.

A particularly intriguing instance of collective emergence of synchronization involves the behavioral displays of male fiddler crabs (*Uca* spp., Decapoda: Ocypodidae). Local populations of fiddler crabs often form large aggregations in intertidal zones, typically in mangroves or sandy beaches of brackish water, where males from burrow-mating species defend their territories and attract females during low tides [10]–[12]. During semimonthly cycles of reproductive activity associated with female receptivity in synchrony with tide cycle and larval release [13], [14], males commonly perform rhythmic waving displays with their greatly enlarged claws (Fig. 1). In addition to courtship displays, these massive appendages also play an important role in other activities, such as advertisement and male-male combat [10], [15], [16], entailing substantial energetic costs [17]–[20], given that these massive claws often approach nearly half of the body mass and four to five times the length of the minor claw used for feeding [16], [21]. While performing waving displays, fiddler crabs often synchronize their behavior with neighboring males both in the presence or absence of a target female [12], [22]–[25]. Although synchronization of communication signals associated with mating behavior is itself common in many other organisms with sound or bioluminescent signals (fireflies [26], [27], katydids [28] and frogs [29]), the synchronized waving displays of fiddler crabs are unique for two reasons: first, they are the only known synchronized visual courtship displays involving reflected light and conspicuous moving body parts [30]. Second, males tend to synchronize more readily with their immediate neighbors, forming partially discrete waving neighborhoods [22]. Many studies demonstrated female preference for males with leading signals, and this leadership was correlated to other physical qualities such as male size and physical strength to maintain high signaling rates [15], [24] yet little is known about the adaptive significance of synchronization itself. One possible explanation is the precedence effect, in which males displaying first with respect to the rest of the group could be particularly attractive to females [25], [31]–[33]. As a consequence, the synchronized courtship observed in the males would likely be an epiphenomenon resulting of the competition for waving first [24], [25], [30], [33]. Alternatively, groups of displaying males could be more attractive to females in general, or to assess each other’s competitive potential [12], [25] or avoid predation [34], in a phenomenon analogous to the selfish herd hypothesis [35].

The formalism commonly used to model behavioral synchronization involves coupled oscillators responding to a mean-field aggregate cue, such as the total sound intensity in chorusing frogs or light brightness in flashing fireflies [36]. In fiddler crabs, however, the field of view of a male tends to be disproportionately affected by signals from its immediate vicinity, leading to the possibility of decay with distance in the degree of synchronization and symmetry breaking when groups of males have non-overlapping waving frequencies. These properties differ from previous spatially explicit synchronization models in ecology, such as those focusing on spatial variation in population density in which spatial coupling among different locations involves spatial migration and/or a limited spatial foraging area [37]–[40].

Fiddler crabs have a 360-degree visual field based on compound eyes made of many ommatidia encircling the tip of the eyestalks held above the carapace. Fiddler crab compound eyes are excellent motion detectors but poor in visual resolution, which is determined by the size and spacing of ommatidia. Better resolution is concentrated near the visual equator all the way around the eyes, aligned with the visual horizon, as an adaptation to their flat environment [12], [41]–[43]. Therefore, the visual field of these crabs is distinctly divided into hemifields below and above the crab’s visual horizon, allowing for discriminating conspecifics from potential predators, which are larger visual objects (such as birds). During displays, the waving apex of conspecifics would penetrate the “predator” or dorsal visual zone and strikingly call the crab’s attention [41]–[45]. However, discriminating predators from conspecifics displayed in the dorsal visual zone is essential to survival and relies on recognition of visual cues such as motion patterns, object size and shape, background, and object contrast [10], [41], [44]–[47]. The distance at which a crab is able to detect and react to the presence of a conspecific can be roughly predicted by the crab’s height, the conspecific size and the visual resolution power, yet this is not easily determined given that a response to the presence of conspecifics is also affected by a set of immeasurable social factors [12], [41], [42], [45], [48].

It is so far not known whether fiddler crabs are able to focus attention on selected visual objects or whether they continually attend to all directions. However, it is clear that the visual system of fiddler crabs is sufficiently elaborate to make waving synchrony possible [22], [23], [28], [47]. In fact, there is evidence suggesting that fiddler crabs direct visual attention: According to Land and Layne [49] male fiddler crabs present preferential orientations toward conspecifics, showing certain parts of the body to intruders and females as a threat or attraction. This could also result from an attempt to align conspecifics with the crab’s highest resolution vision [50], possibly indicating that they direct attention to approaching conspecifics and establishing a narrower angle of attention in the 360-degree visual field. However, no study to date has identified a specific angle of concentrated attention toward conspecifics and there is still not enough evidence to prove its existence.

In this study we investigate a spatially explicit model of synchronization in fiddler crab waving displays. We chose the logistic map as the basis for our model because it has a rich dynamical behavior (from periodic orbits to chaos) and it has already been extensively studied [51], [52], including coupled logistic map models [53]–[58]. Although selected visual attention in several different directions at once is discarded in this first attempt to model waving synchrony, we approach two extreme situations: a limited angle range, and *360*° attention vision. The model also considers that all individuals are equivalent, meaning that all of them have the same physical strength and visual power, and no background interference is considered. Despite its simplicity, our model was able to successfully reproduce many of the main features of fiddler crab waving behavior, such as local synchronization and the formation of synchronized neighborhoods through symmetry breaking.

## Materials and Methods

### 2.1 Logistic Map

**Figure 2:**
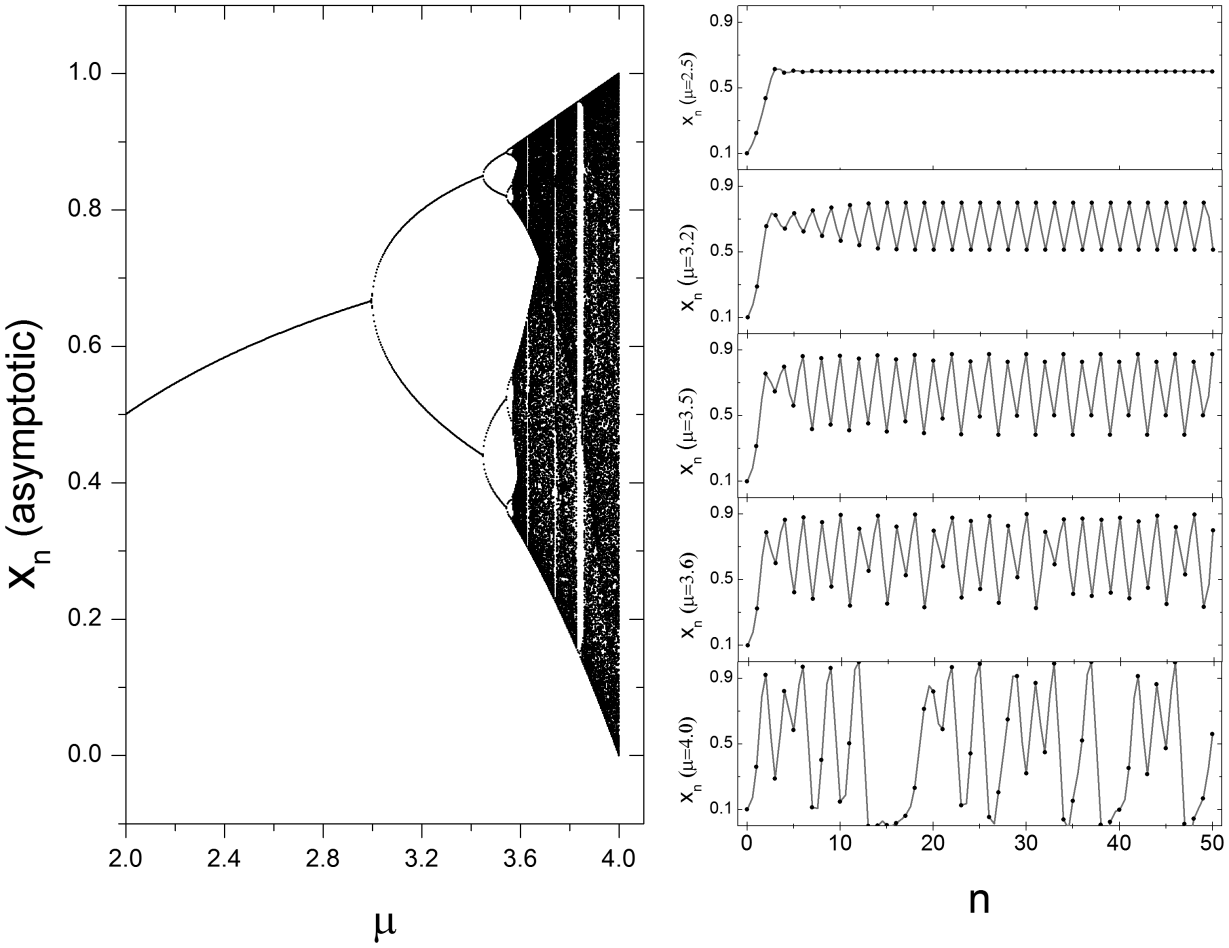
Logistic map. Left: Bifurcation diagram shoes the asymptotic value of xn as function µ. Right: Temporal evolution for five different values of *µ*: *µ = 2.5* (stable fixed point), *µ = 3.2* (period two), *µ = 3.6* (chaos) and *µ = 4.0* (chaos). In all cases the initial conditions were set on *x_0_ = 0.1*.

The logistic map is well known [51], [52] because of its rich dynamics emerging from a simple equation:
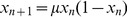
(1)where *x_n_* is the variable at time *n* and *µ* is a positive constant. If *0<x_n = 0_<1* and *1<µ<4*, the sequential variable values is always *0<x_n_ <1*. For *1<µ<3*, the fixed point, *1−1/µ*, is stable, which means that for any *x_0_* value, *x_n_* evolves to the fixed point. For *3*≤*µ*≤*4*, the fixed point is unstable and the asymptotic dynamics of *x_n_* depends on *µ*, such that a bifurcation takes place when *µ = 3* and *x_n_* converges to period two dynamics (*i.e. x_n_* oscillates between two values). When *µ = 3.45*, there is another bifurcation, leading to a period four dynamics. As *µ* increases, successive bifurcations arise until the point (*µ* = 3.57), when the period of the system approaches infinity, leading to the onset of chaos. Another remarkable dynamics present in the logistic map is the presence of periodic dynamics for some values of *µ* greater than *3.57*. The larger periodic window occurs when *µ* is around *3.83*, where the dynamics is a period three (Fig. 2).

Regarding fiddler crab waving displays, *x_n_* can be interpreted as a claw displacement at time *n*. A period two dynamics means that the crab takes two units of time to complete a full wave, period four means that it takes four units of time, and so on. The chaotic regime would mean that the crab never wave exactly in the same way. Although this seems to be unrealistic at first, this regime can be interpreted as just a small variation on the displacement in a given time. For example, compare the temporal evolution when *µ = 3.2* with *µ = 3.6* (Fig. 2): both cases led to up and down sequential movements, yet the amplitude is not exactly the same.

### 2.2 Model Description

We represent space by a two dimensional regular lattice with *LxL* sites. Each site corresponds to an area around the male’s burrow. We refer to it as its home position and male displacements from this area are not considered in this version of the model. The claw movement of each individual is modeled by the coupling logistic map:
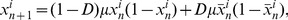
(2)where



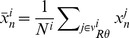
(3)


**Figure 3:**
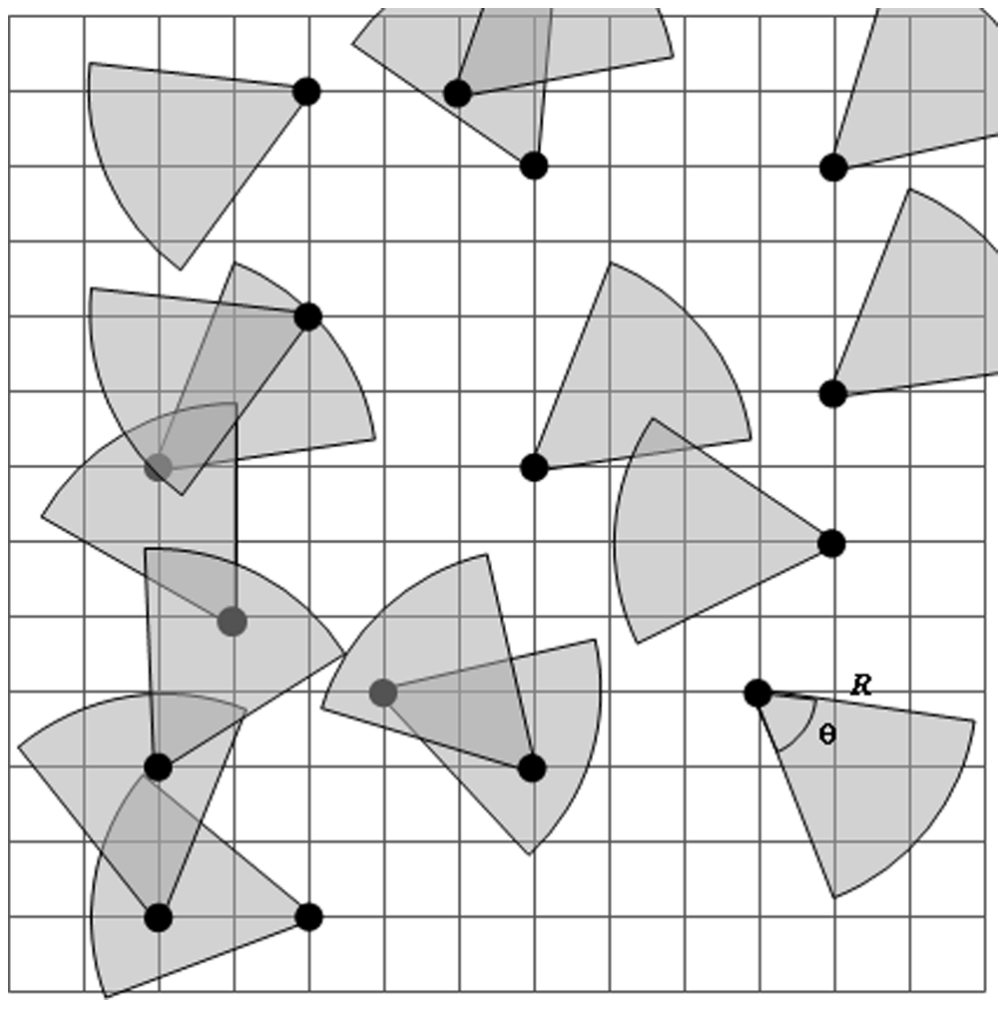
Illustration of male spatial distribution with random visual attention orientations. The intersections of the grid represent all possible home positions and the black dots represent all occupied sites. Filled gray area shows the field of attention, defined by *R* and *θ*.

The variable 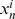 indicates claw displacement at time *n* of the individual whose home position is in site *i*. Given that there is only a single crab per site, the subscript *i* identifies both the home site and the individual male. The first term of Eq. (2) models claw movement without the influence of other neighbors. The second term represents the aggregate influence of other males on a crab’s own claw movement. Variable 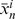 thus represents the average of the neighbor’s claw displacements, where *N^i^* is the number of individuals in the field of attention of individual *i* (Eq. 3). The field of attention is defined as a section whose area is 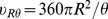, where *R* and 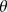 are the reach and the angle (in degrees) of attention, respectively. Given that, to date, no study on the visual ecology of fiddler crabs has been able to find a definite angle of attention, we tested different 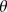 values, including 360°. The parameter 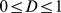 is the coupling constant: the higher its value, the stronger the influence of other individuals on the wave of individual *i*. When *D = 0*, we have the well-studied logistic map [51], whereas if *D>0* the system becomes more complex and its dynamics cannot be predicted analytically. Our model also allows for varying crab density, such that the regular grid is randomly occupied by *ρL^2^* individuals, where *L* means the lattice width and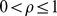. The model considers absorbing boundary conditions, *i.e.* individuals that have their field of attention beyond the grid are not influenced by other individuals. Fig. 3 illustrates the two dimensional grid with the individuals and their set fields of attention.

We assume that the individuals stay in their home position and their set field of attention is constant. We provide two alternative models to assess the influence of the orientation of the male’s field of attention on their synchronization: (1) *M1*: The orientation of a male’s field of attention is random with respect to the presence of other males; (2) *M2*: The orientation of a male is preferentially directed such that the number of males in its field of attention is maximized. If two equally favored alternative fields of attention are present, the male chooses one of them with equal probability. After all fields of attention are set, the iteration of Eq. 2 starts. We also approach a global field of attention (θ* = 360*°), which reorientation does not make sense and *M1 = M2*. A unique and small angle of attention and a maximum one (when *θ = 360*°) are two extreme possibilities within the crab possible range of angles.

We also have studied other scenarios in which the model includes additional assumptions. Among them, it is interesting to point out two: (a) We modified *M2* considering that in the presence of more than one preferential field of attention, an individual chooses the direction which the average movement of the visible individuals looks more similar to its own (that is 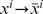); (b) We considered that the coupling decreases with distance. As consequence, Eq. (3) was treated as a weighted average, 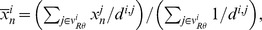 where *d^i,j^* is the distance between *i* and *j*. Although both additions seem to make the model more realistic, they do not qualitatively change the predictions. Therefore, we restrict our discussion to *M1* and *M2*.

### 2.2 Model Analysis

In order to quantify synchrony between individuals, we calculate the average correlation 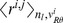 between individual *i* and the neighbors *j* within its field of attention, 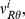 during a time lag *n_l_*:
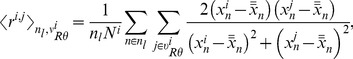
(4)where 
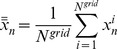


*N^i^* is the number of individuals in the field of attention of individual *i*, and 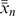
is the global average, where the sum takes into account all individuals in the grid, *N^grid^*. In order to obtain a convergent global correlation value *<r>*, we compute a global average obtained from *s_t_* simulations and for the individuals *i* that are inside a smaller grid of size *L_c_xL_c_*, centered in whole grid (The restriction over *i* was made to minimize boundary effects):
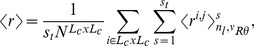
(5)where 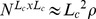 is the number of individuals in the centered grid.

### 2.3 Analytical Insights

To get some insights about the coupling logistic model, let us first consider a special case with only two individuals. In this case, there are two possibilities only: *(a)* bidirectional coupling, where both individuals follow each other; *(b)* leader-follower coupling, where only one individual follows to the other, whereas the latter one has no influence on the behavior of the leader. This coupling structure is also known as master-slave coupling [58]. As detailed below, these two possibilities result in different synchronization conditions.

#### 2.3.1 Bidirectional coupling: •↔•

This simplified version has already been studied previously [53]–[55] for small values of *D(D<0.5)*. Here we consider the full range of *D (0<D<1).*

By considering two individuals, *i* and *j*, that influence each other, Eq. (2) is simplified to:
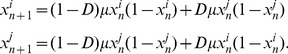
(6)


In terms of the amplitude difference, 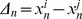, Eq. (6) can be greatly simplified. We obtain:
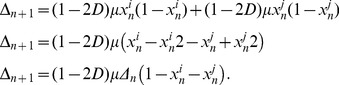
(7)


Observe that the two individuals will synchronize if the absolute difference amplitude diminishes over the time, 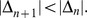 Here we can already infer that, if the coupling parameter is *D = 0.5*, the two individuals synchronize in a single time step. Since the logistic map limits 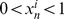, we have that 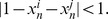 As consequence, we can write down the following restriction for synchrony:
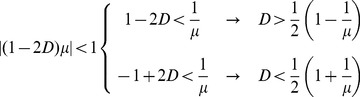
(8)

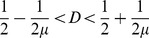
(9)


This condition is sufficient for synchronization, but it is not necessary since 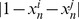 can assume values smaller than 1. For this situation we expect a wider range of *D.* In fact, the study by Lloyd [55] considered *0<D<0.5*, and observed numerically that, for *µ = 3.2* and *D>0.058*, this system always synchronizes.

#### 2.3.3 Leader-follower coupling: •→•

If only one individual pays attention to the other, Eq. (2) simplifies to:
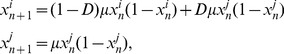
(10)where the dynamics of *i* has no influence over *j*. Analogous to the previous case, we rewrite the above equation in terms of the difference amplitude, 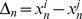
and obtain



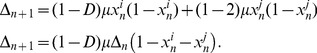
(11)The condition for synchronization is then:
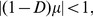
(12)and results



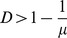
(13)Again, this condition is sufficient for synchronization, but it is not necessary since 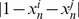 can assume values smaller than 1.

**Figure 4:**
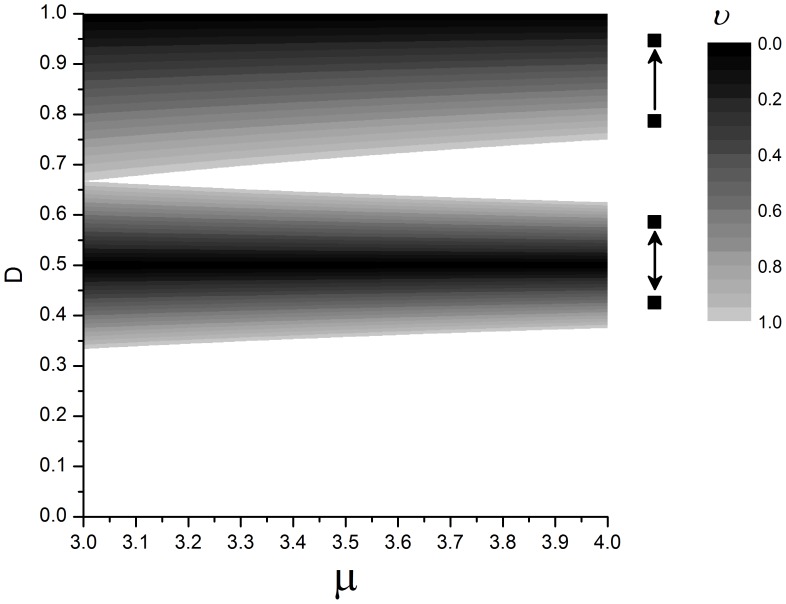
Analytical restriction for two individuals, obtained by Eq. (9) and Eq. (13). The contour plot shows how fast the system becomes synchronized: the darker is the plot, the faster it occurs. The upper contour plot refers to the follower-leader coupling, and the contour line values correspond to *ν = (1−D)µ.* The lower contour plot refers to the case where the coupling is bidirectional, and the contour lines values correspond to*ν = |(1−2D)| µ.*

The full model proposed here considers not only two, but many interacting individuals. However, as we will see, these two simple interactions, that impose different restrictions, are present in the full network and can help us to understand the synchronization dynamics of the complex model. Both conditions, Eq. (9) and Eq. (13), are plotted in Fig. 4.

## Results

**Figure 5:**
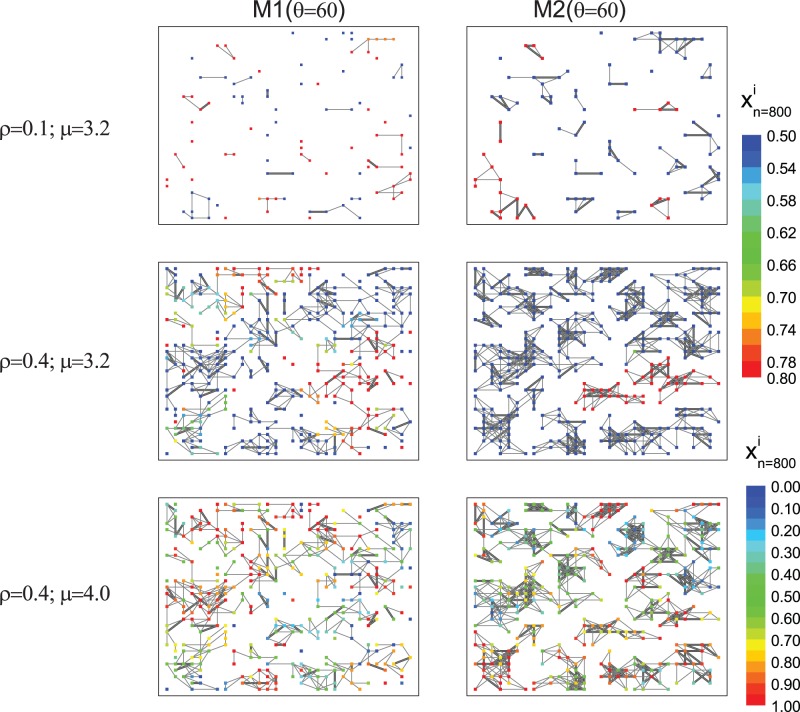
(Color online) Spatial distribution at time *n = 800* for *M1* and *M2*. Each dot represents one individual, whose claw displacement value,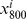, is identified by the color scale on right of the graphs. Links shows the presence of interactions. Narrow lines means that the interaction occurs in just one direction (the interaction direction is not plotted) and wide lines means bidirectional interaction. The imposed density values, *ρ = {0.1, 0.4},* and the logistic map values, *µ = {3.2, 4.0}* are shown on left of each graph. For all graphs the coupling constant value was set on *D = 0.5*. In order to contrast the spatial interaction network predicted by *M1* and *M2*, in these graphs the spatial positions of the crabs are the same (for a given density), the unique difference is the orientation.

For all simulations presented here, initial conditions were set as: reach of attention *R = 3*; lattice width *L = 30*; and lattice width for the correlation calculation *L_c_ = 10*. We ran the simulations for different values of *µ* (logistic map constant), *D* (coupling constant), *ρ*(density) and two alternative breadths in the angle of attention, *θ = {60*°,*360*°*}*.We tested different *L*, *L_c_* and two other *θ* values (30° and 90°) and found that they had no qualitative effects on the investigated results. *F*or *θ* smaller than *360*°, we tested either random field of attention orientation (*M1*) and preferential field of attention orientation toward neighbors (*M2*), whereas when *θ = 360*° both model version are equivalent. Each simulation included at least *s_t_ = 500* replicates and a time lag *n_l_ = 500* to obtain a convergent global correlation value. Throughout the text, we consider as a leader the individual that does not follow any other male (because there are not any individual in its field of attention), and as a follower the individual that follows another male, regardless if the last one is also a follower or a leader. Bidirectional coupling refers to two individuals following each other. Examples of the spatial organization and claw displacements, when *θ = 60*°, for *M1* (random field of attention orientation) and *M2* (preferential field of attention orientation toward neighbors) are shown in Fig. 5. Under low density, the whole grid is composed of unlinked groups, each having few coupled individuals. The bidirectional coupling can occur in both models, as long as two individuals overlap their field of attention. On the other hand, follower-leader coupling is only present in *M1* (if *θ<360*°), whereas, for *M2*, the reorientation leads to bidirectional coupling. As consequence, bidirectional coupling is more frequent in *M2* than *M1*. As density increases, the whole grid becomes more connected, yet field of attention reorientation results in more cohesive subgroups (Fig. 5).

**Figure 6:**
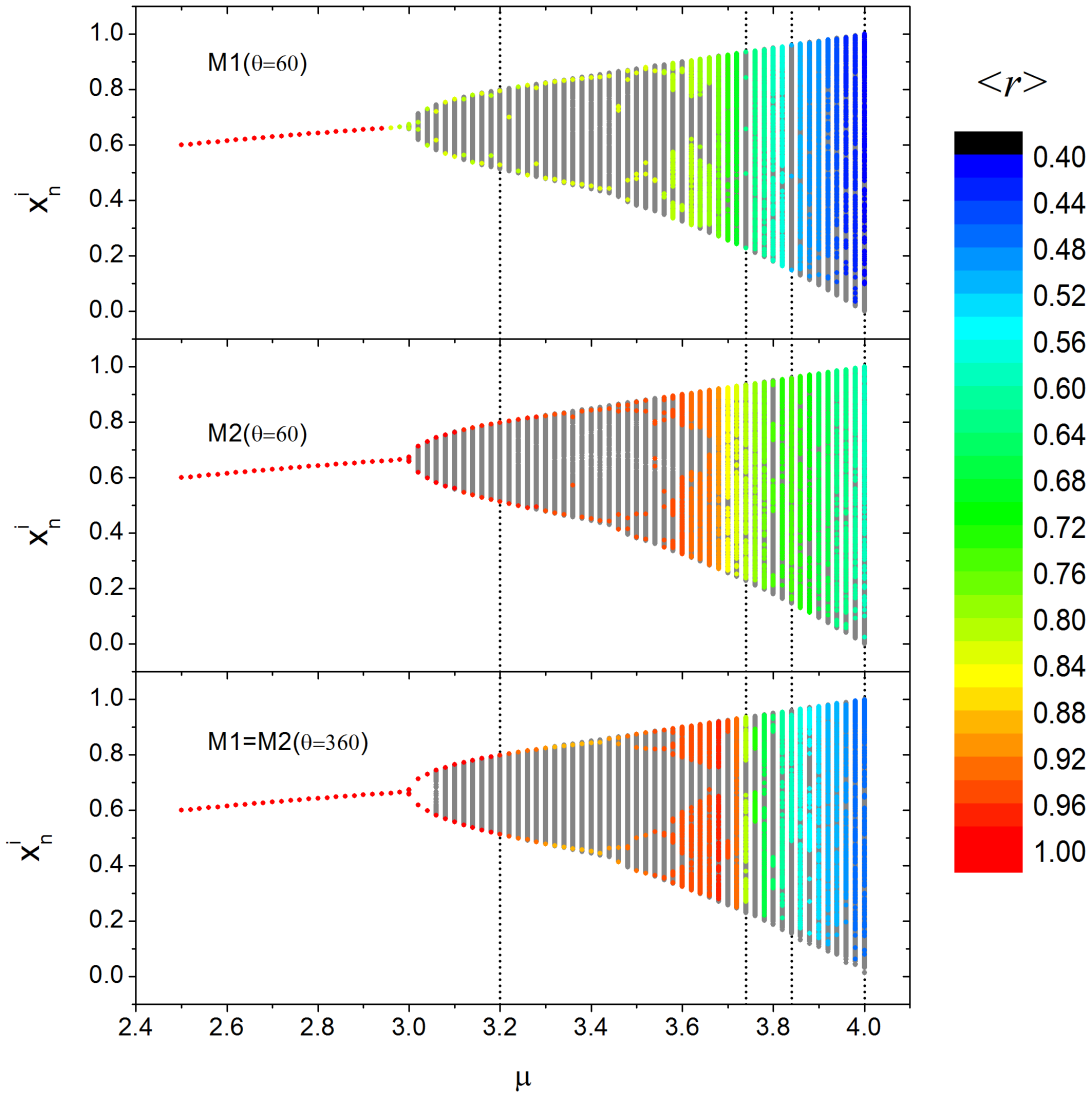
(Color online) Bifurcation diagram for *M1* and *M2* considering *ρ = 0.4* and *D = 0.5*. Gray dots show all possible claw displacement values assumed by all individuals. Colored dots show one possible claw asymptotic displacement values (assumed by one individual) for 800<*n<1000.* Colored scale refers to the global correlation value, *<r>*. For *µ<3* both models predict a stable fix point (gray dots are behind to colored dots). Vertical black dot lines highlight the cases where *µ = {3.2, 3.74, 3.84, 4}.* The first and the last values are the ones set in Figs. 5 and 7. The other two values have, respectively, five and three period orbits only for *M1* model (as predict by uncoupling logistic map).

By following the dynamics of any individual in the grid and constructing the bifurcation diagram (Fig. 6) we observed a clear correspondence between both models and the uncoupling logistic map [52], such as the presence of stable fix point for *µ<3.0*, period two orbit for *3≤µ<3.45*, and the following cascade of period doublings. We have not checked if the coupling models also lead to chaos, yet given that their bifurcation diagrams are so similar to the uncoupling logistic map, we believe that chaos could also be present for *µ>3.57.* Odd period orbits were observed only for *M1 (θ = 60*°) when *µ = {3.74, 3.84}*, in agreement with the uncoupling logistic map (compare Fig. 2 with Fig. 6). It is interesting to point out that, for the periodic orbits, all individuals have the same period and most of them have the same orbit amplitude as the uncoupled logistic model (Fig. 5), however all intermediate amplitude values can also occur (Fig. 6). For example, when *µ = 3.2*, each individual follows a period two dynamics, but not all claw displacements assume the same pair of values. Due to the period two dynamics, the spatial pattern present in Fig. 5 is exactly the same for any time *n* multiple of 2 and far from the transient. Another interesting result indicated in Fig. 6 is that *M1* loses synchrony much faster than *M2*. For example: when the dynamics change from a stable fix point to a period two orbit, *M1* global correlation decreases from 1 to around 0.8, whereas *M2* maintains a high correlation value. This figure also shows that the increase of degree of attention is not always more favorable for synchrony: for *µ* around *3.35* or greater then *3*.7, a small degree of attention with reorientation (M2, *θ = 60*°) facilitates the synchronization more than *θ = 360*° *(M1 = M2).*

**Figure 7:**
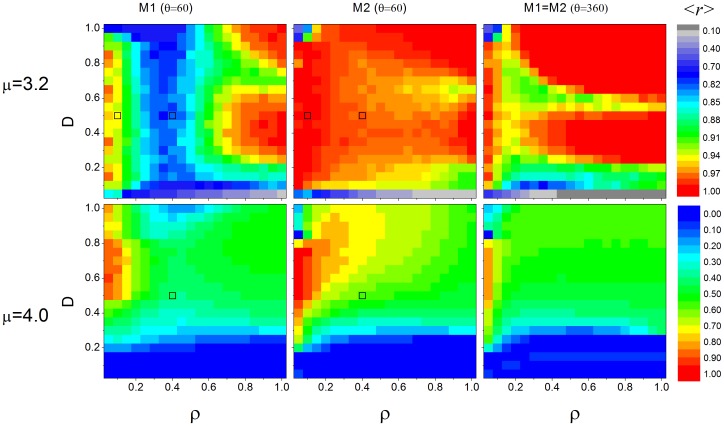
(Color online) Phase diagrams. The graphs show the global correlation *<r>*, Eq. (5), for *M1* and *M2* models and two values of logistic map constant, as function of coupling constant, *D*, and the density of individuals, *ρ*. The global correlation value corresponds to the color scale on the right of the graphs. The open black squares highlight the parameters present in Fig. 5.

**Figure 8:**
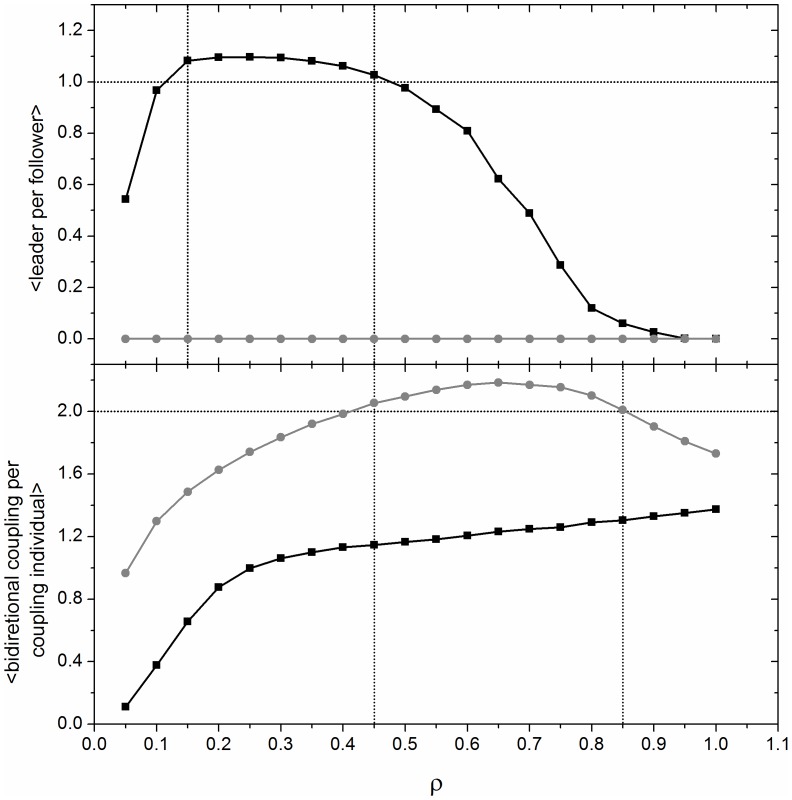
Consequence of spatial organization for *M1* (black dots) and *M2* (gray dots) obtained by the average of 5000 initial conditions for each *ρ* value. Above: average number of leader per follower. Observe that, for *M1* and *0.15≤ ρ≤0.45*, there is more than one leader per follower, causing the asynchronicity observed in Fig. 6. As in *M2* the individuals rearrange themselves in order to maximize the number of individuals in their field of attention, there is not any leader present. Below: average number of reciprocal couplings by individuals that makes at least one reciprocal coupling. Observe that, for *M2* and *0.45≤ ρ≤0.85*, there are more than two reciprocal couplings per individual, causing a decrease of synchrony, as observed in Fig. 7 for *µ = 3.2*.

Global correlation value, *<r>*, are shown in Fig. 7 for various conditions (*θ = 60* (for both models) and *θ = 360*° (*M1 = M2*), assuming *µ = {3.2, 4.0}* and different combinations of coupling constant, *D*, and density, *ρ*). Given that chaos is characterized by its high sensitivity to initial conditions, we could expect that, if the individuals have a chaotic claw movement, the synchronization would be lower than if they have a periodic movement. In general, Fig. 7 shows that this expectation is true, except for low density, where both *µ* values result in high global correlation value. In fact, Eq. (9) and (13) and Fig. 4, predicts that perfect synchronization (*<r> = 1*) can occur for all *µ* values. As we have already pointed out, the similarity of the coupling models to the case of two individuals studied analytically is higher under low density. In this condition, synchronization is independent of *µ* for *M1* when *D* assumes high values (because most of two coupled interactions are of the “leader-follower” type) and for intermediate values of those parameters for *M2* and *θ = 360*° (because most of two coupled interactions are bidirectional), in agreement with the analytical predictions. In addition, the only situation where *M1* synchronizes more intensely than *M2* is for low density and high coupling constant values. As density increases, the grid becomes more connected and synchronization is more sensitive to *µ*. However, for *µ = 3.2* and high density, Fig. 7 shows that, in both models, synchronization is not as strong for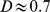, as predicted analytically (Fig. 4). Moreover, we can infer that for high densities, synchronization works like a follower-leader coupling if *D* assumes high values (upper right graph region), or, if *D* around *0.4*, the network can still be well synchronized, meaning that the influence of part of the whole group on another is bidirectional. Although the increasing of density results in a much more complex spatial network (in comparison to the analytical version) this result shows that the simplest spatial description can still be very informative in relation to the complex spatial network. We have tested if this pattern is also present for other *µ* values, which the asymptotic dynamics for the uncoupled logistic map corresponds to period two (3.4), four (3.46), eight (3.55), sixteen (3.565), and chaos (3.6 and 4.0). We noticed that this valley is smoother as *µ* increases, and that it disappears for period eight, showing that the analytical prediction fails as the dynamics become more elaborated. Fig. 7 exemplifies the case where *µ = 4.0* and there is no restriction for 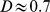. As shown in Fig. 6, when *θ = 360*°, synchronization is not favored in all scenarios. In general, *M2* promotes stronger synchronization under low densities (*ρ<0.4*), regardless of *µ*. When *µ = 4.0, M2* has a higher global correlation value for all densities if *D>0.5* and a similar value for *D<0.5* Another intriguing pattern present in Fig. 7 is that, although synchronization can be high for low and high densities, it can be fairly low for intermediate densities as well. For *M1* this phenomenon occurs regardless of *µ* value, meaning that there is at least one spatial network property that is present in low and high densities that is not present for intermediate densities. In order to further explore this phenomenon, we counted, for each considered density value, how many leaders each follower has. Then we calculated the average in the *L_c_xL_c_* central grid over 5 thousand spatial networks. We observed that, for *0.15≤ρ≤0.45*, each follower has, on average, more than one leader (Fig. 8). In fact, if two leaders are not synchronized and the behavior of a follower is influenced by both, it is impossible that these three individuals will synchronize. For *M1* and *0.10≥ρ≥0.50*, the number of leaders that each follower has is, on average, less than 1, meaning that not all network configurations had a leader, and when they did occur, they tended to have only one follower. The increase of the number of leaders around *ρ≈0.3* is a consequence of a spatial limitation. To explore this result further, one can imagine a region of empty space that receives individuals at random. For low densities, the probability of having one individual in the field of attention of another is low. As we include more individuals the number of leaders increases. However, for an intermediate density, those individuals that were leaders at low densities now have new individuals in their field of attention (and thus become followers). In this hypothetical example, the probability of new individuals not having any individual in their field of attention also decreases as the density increases. When the density is at its maximum value, it is impossible to have any leader because all individuals necessarily see others. On the other hand, for *M2*, there is no leader for any density value because a potential leader reorients its field of attention and connects at least with its follower. However, there is also a valley present in Fig. 7 for *M2 µ = 3.2*, when *ρ* is around 0.6. We have observed that this valley is present for *µ<3.5* (that corresponds to the periodic orbits). Similarly to the previous analysis, we looked for a network characteristic dependent only on density. We found that the number of individuals with at least one bidirectional coupling increases in this valley (Fig. 8). For this region, the average number of bidirectional couplings per individual is higher than 2. Contrary to the previous analysis, it is unclear whether two or more bidirectional couplings reduce synchronization. In fact, this effect is present just for periodic orbits and the loss of synchronization is fairly smooth (Fig. 7, *µ = 3.2*). We also counted the average number of individuals in the unlinked groups as function of density and confirmed that group size increases with density, without notable distribution changes in the valley.

## Discussion

In the present study, we provide the first effort to model explicitly the phenomenon of synchronization of male waving displays in fiddler crabs. Our study is particularly novel given that two of the main characteristics of models – the lack of migration and the restriction of spatial interactions to circular sectors – have not been explored hitherto in the biological literature. In particular, we explored two alternative spatial models for the synchronization of waving displays with limited attention angle: either with random field of attention orientation (*M1*) or with males preferentially orienting their field of attention toward their neighbors (*M2*). We have also approached *360*° attention vision (*M1 = M2*). Although synchronization was possible for all scenarios both *M1* and *M2*, the possibility of limited field of attention with reorientation had a profound influence on the resulting behaviors.

First, *M2* led to increased clustering in interaction networks and more defined synchronization neighborhoods than *M1* and *M1 = M2* (360°) (Fig. 7). This indicates that directing attention to the whole 360° visual field at a time leads to a decrease in the level of synchrony. In natural conditions, it is common for small groups of synchronic waving males to form around a passing female while other males outside the group wave out of synchrony [22], [24], [25]. The formation of groups is evidence that the visual information coming from it (waving males) is the one that triggers the response of the observer to wave in synchrony, in contrast to the visual information coming from waving males from outside the synchronic group [59]. Therefore, despite evidence that fiddler crabs respond to certain visual objects over others, this is not prove of the existence and extent of angles of attention. Our results suggest that fiddler crabs have selective attention, given that M2 facilitates synchronization. However, the predictions from this study together with the available data on fiddler crab visual systems tested against empirical data should clarify the complexity of surrounding perception particularly toward conspecifics during synchrony. Determining the extent of visual attention is important to further understand the formation of waving neighborhoods in fiddler crabs.

Synchronization was considerably more robust in *M2* than in *M1 = M2* and *M1* with respect to increases in *µ,* in other words waving complexity (Fig. 6). In nature, it is known that some species increase wave complexity and conspicuousness when seeing an approaching female [e.g. 60, 61]. However, according to the data shown in this study, synchrony decreases with the increase in display complexity. Therefore, species that respond by increasing both complexity and conspicuousness such as *Uca leptodactyla* (Perez personal observation) and *U. perplexa* [25], [61], probably face greater challenges in attracting mates. If cautiously analyzed, waving complexity (*µ*) is an interesting pattern given its potential to be tested against the diversity in standardized waving displays found in the genus [10], [62]–[66]. Therefore, future research on modeling waving synchrony could adjust *µ* according to the studied species.

Moreover, *M1*, *M2* and *M1 = M2* also show different predictions with respect to the relation between density (*ρ)* and the level of synchronization (*r*), which tends to be much lower at intermediate densities in *M1* (Fig. 7). In order to unveil the influence of density on synchrony, empirical data with density manipulation of synchronic males is needed. Investigations of the type are already been conducted and should reveal the unfavorable densities for synchronization. Additionally, coupling (*D*) is another factor that showed unexpected patterns where its increase does not always result in higher synchrony (*r*). In fact, the influence of other waving crabs in field might be a highly complex and variable aspect [67], [68].

Given the 360° fiddler crab’s visual field [12], [41]–[43], *M1* is less likely to happen in nature, especially regarding a fixed field of attention that is randomly oriented. Additionally, in contrast to *M2* and *M1 = M2, M1* is the only case presenting the leader-follower coupling where the leader does not see another crab (fig. 8; see results). Fiddler crabs have an interesting framework where females likely chose males that wave first in a synchronic group as an indicator of male quality [24], [25], [30], [32], [33], [67]. Considering that this very unlikely visually limited situation is set, maybe due to physical visual barriers, where one male waves alone without seeing the others that follow him, a passing female which could assess all crabs in this group would chose the leader, even if all males in the synchronic group have same physical strength and stamina. Selecting the leader in this particular case would not mean that the female is choosing the fittest.

The remarkable simplification of the model is to assume that all males are equivalent. Small variations in the wave displacement (*μ*) and in the influence of other individuals on the wave (*D*) could encompass intraspecific variations and better represent waving leadership. Although is very unlikely that attention is focused on certain sectors, defined by angles in this study, the model does not consider that the individuals move through space, so it is reasonable to assume that it serves effectively as attention towards objects.

Recent years have witnessed a strong interest in understanding collective behaviors in animal groups, such as ant colonies, bird flocks and fish schools [7]. The synchronization in waving displays in fiddler crabs provides an ideal model system to investigate collective behaviors on a simple two-dimensional space, given the relatively small scale of the phenomenon and the simplicity in the individual behaviors. Moreover, the large number of species and the remarkable interspecific variation in waving displays themselves [65], [66] indicate the possibility of phylogenetic comparisons that would allow for a window into how self-organized behaviors can evolve as species adapt to varying ecologies.
